# A Novel TiZrHfMoNb High-Entropy Alloy for Solar Thermal Energy Storage

**DOI:** 10.3390/nano9020248

**Published:** 2019-02-12

**Authors:** Huahai Shen, Jianwei Zhang, Jutao Hu, Jinchao Zhang, Yiwu Mao, Haiyan Xiao, Xiaosong Zhou, Xiaotao Zu

**Affiliations:** 1 Institute of Nuclear Physics and Chemistry, China Academy of Engineering Physics, Mianyang 621900, China; huahaishen@caep.cn (H.S.); yiwmao@gmail.com (Y.M.); zlxs77@126.com (X.Z.); 2 School of Physics, University of Electronic Science and Technology of China, Chengdu 610054, China; jianweizhang19@163.com (J.Z.); hujutao_uestc@sina.com (J.H.); xtzu@uestc.edu.cn (X.Z.)

**Keywords:** high-entropy alloy, thermal energy storage, crystal structure, phase transformation, X-ray diffraction

## Abstract

An equiatomic TiZrHfMoNb high-entropy alloy (HEA) was developed as a solar thermal energy storage material due to its outstanding performance of hydrogen absorption. The TiZrHfMoNb alloy transforms from a body-centered cubic (BCC) structure to a face-centered cubic (FCC) structure during hydrogen absorption and can reversibly transform back to the BCC structure after hydrogen desorption. The theoretical calculations demonstrated that before hydrogenation, the BCC structure for the alloy has more stable energy than the FCC structure while the FCC structure is preferred after hydrogenation. The outstanding hydrogen absorption of the reversible single-phase transformation during the hydrogen absorption–desorption cycle improves the hydrogen recycling rate and the energy efficiency, which indicates that the TiZrHfMoNb alloy could be an excellent candidate for solar thermal energy storage.

## 1. Introduction

Metal hydrides have been considered to be one of the most important solar thermal energy storage materials due to their advantages of high energy density, reversibility and thermal stability [1,2,3,4]. The solar energy is stored through the dissociation of metal hydride into its components and the heat energy is released again during the hydrogen adsorption of the metal [1,2,3,5]. The development of novel metal hydride materials with high storage capacity [6], good thermal stability [7,8] and reversible phase transformation during the hydrogen absorption–desorption cycle [9] is crucial for enhancing the applications of solar thermal energy. Ti, Zr metals and their alloys (ZrCo [10,11], Ti-Mo [12] and Ti-V [13], etc.) are important material systems for hydrogen storage due to their high storage capacities and low equilibrium pressures at room temperature (RT) [9]. However, the low oxidation resistances of Ti-H [9] and Zr-H [14] systems, the severe lattice expansion during hydrogenation and the disproportionation phenomenon of ZrCo-H restrict their applications in solar thermal energy storage. It has been proved that the adding alloying elements into Ti and Zr metals was an effective way to regulate their hydrogenation properties [13]. The lattice parameters of Ti alloys decrease with an increased content of doping elements V [13] and Mo [12]. However, the addition of alloying elements might induce the disproportionation of the alloy hydride materials and significantly decrease the hydrogen recycling rate [10].

High-entropy alloys, which were proposed by Yeh et al. [15] in 2004, were designed using a completely new concept and synthesized by mixing five or more metallic elements to form a single phase alloy with a BCC or FCC crystal structure [8,16,17,18,19,20,21]. Recently, HEAs have received significant attention in the hydrogen storage field due to their excellent hydrogenation properties, including superior hydrogen storage capacity and good thermal stability [22,23,24,25,26,27]. The lattice distortion feature of an HEA [16] provides more interstitial sites that can accommodate hydrogen atoms [22]. Sahlberg et al. [22] proposed that a TiVZrNbHf alloy has a maximum hydrogen storage capacity of 2.7 wt.% and has a high thermal stability of 500 °C for its hydride [8,22]. Kunce et al. synthesized ZrTiVCrFeNi [26], TiZrNbMoV [27] and La-Ni-Fe-V-Mn [20] HEAs using the laser engineered net-shaping technology and studied the correlation of their hydrogenation performance with annealing temperature, fabrication parameters and chemical composition, respectively. Zepon et al. [25] reported that a MgZrTiFe_0.5_Co_0.5_Ni_0.5_ alloy processed by high-energy ball milling under argon and hydrogen atmospheres was capable of absorbing up to 1.2 wt.% of hydrogen. Chen et al. [24] systematically studied the correlation between hydrogen storing behaviors (maximum hydrogen absorption, lattice expansion during absorption and the hydride formation enthalpy) and the alloying element concentration of Cr_u_Fe_v_Mn_w_Ti_x_V_y_Zr_z_ alloys.

However, most of the reported HEA systems that used metal hydrides are composed of multiphase [26,27] or multiple products generated after hydrogen desorption [22], which might affect the hydrogenation performance and reliability of their corresponding HEA hydrides. Because the H-absorbing elements Ti, Zr and Hf have the same hexagonal close-packed (HCP) structure [9,14], it is expected that mixing Ti, Zr and Hf metals would result in the formation of single-phase solid solutions with a HCP structure. The H-desorbing elements Mo and Nb might be good for regulating the alloy crystal structure to provide more interstitial sites for accommodating H atoms as they have a BCC structure. The addition of Mo and Nb elements is also beneficial as it improves the oxidation resistance of the alloy. Based on the criterion suggested by Zhang et al. [16], a novel HEA alloy consisting of Ti, Zr, Hf, Mo and Nb metals was designed and fabricated in this work. The hydrogenation performance and the phase transformation property of this new TiZrHfMoNb alloy were also studied.

## 2. Materials and Methods

The equiatomic Ti, Zr, Hf, Mo and Nb metals were weighed to synthesize the TiZrHfMoNb alloy by the arc-melting method. All of the row materials were in the shape of small particles with a size of 1–10 mm and had purity of 99.99%. The argon gas with a purity of 99.99% was filled into the arc furnace to a level of 0.5 bar after the chamber was evacuated to a base vacuum pressure of 5 × 10^−3^ Pa. The Mo, Nb and Hf metals with higher melting temperatures were put on the top layer and completely melted by increasing the arc current up to 600 A. Subsequently, the melted Mo, Nb and Hf elements were mixed with the bottom layer Ti and Zr metals to form an alloy ingot after the arc current was further increased to 800 A. The alloy ingot was re-melted five times to improve the homogeneity of the elemental distribution. The as-obtained TiZrHfMoNb alloy was grinded into micro-sized powders in the agate before the hydrogenation experiments and structural characterization using X-ray diffraction (XRD).

The hydrogenation of the TiZrHfMoNb alloy was carried out in a self-made Sievert’s type apparatus, which sustains up to 50 bar hydrogen pressure at the highest temperature of 600 °C. The in situ heating XRD characterization was performed using a X’Pert PRO MPD (PANalytical B.V., Almelo, The Netherlands) equipped with a XRK-900 environmental chamber working at 45 kV and 40 mA. The typical scanning parameters included 2*θ* of 25°–75°, step size of 0.013° and count time of 50 s. The XRD patterns were acquired once reaching the target temperature from RT to 600 °C with increasing intervals of 50 °C. Thermal desorption spectroscopy (TDS) measurements was obtained from RT to 1000 °C at a heating rate of 10 °C in a Netzsch STA 409C facility (Netzsch, Selb, Germany). To avoid severe oxidation of the TiZrHfMoNb sample, the in situ heating XRD experiment and TDS test were performed in the inert gas flowing environment of helium and argon, respectively. Bright-field images (BF) and selected area electron diffraction (SAED) patterns were obtained to determine the crystal structure of the TiZrHfMoNb alloy before and after hydrogenation using a FEI Tecnai F30 (FEI, Hillsboro, OR, USA) transmission electron microscope (TEM) working at 300 kV. The TEM specimens were prepared by grinding the alloy into powders in the agate and the particles with nanometer sizes were selected for TEM analysis.

The calculations were carried out within the density functional theory (DFT) framework as implemented in Vienna Ab Initio Simulation Package (VASP) [28]. Projector augmented-wave pseudopotentials were used to describe the interaction between ions and electrons and the exchange-correlation effects were treated by the Perdew–Burke–Ernzerhof (PBE) functional within the generalized gradient approximation [29]. For BCC and FCC structures, a 5 × 2 × 1 and a 5 × 1 × 1 supercell was employed, respectively. During the calculation, a 4 × 4 × 4 k-point sampling and a cutoff energy of 600 eV were used.

## 3. Results and Discussions

**Figure 1:**
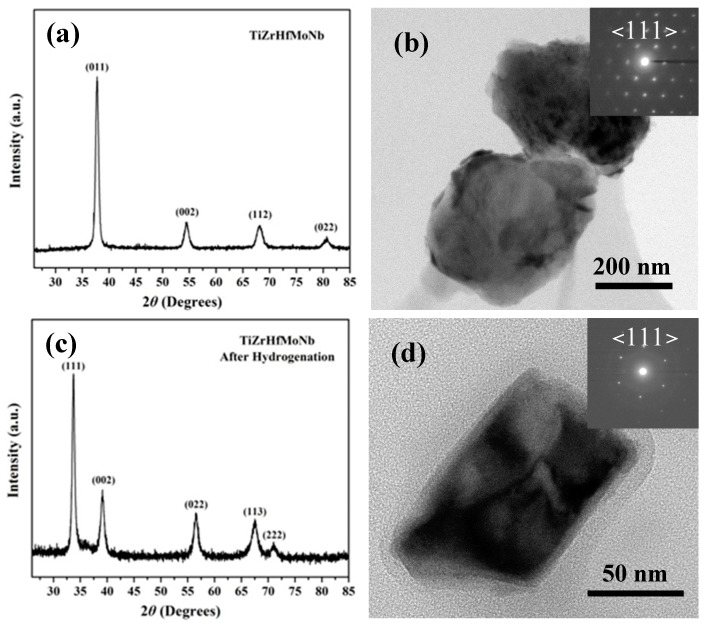
XRD pattern (**a**) and SAED pattern (**b**) of the as-obtained TiZrHfMoNb alloy and XRD pattern (**c**) and SAED pattern (**d**) of the TiZrHfMoNb alloy after full hydrogenation (five hydrogen absorption–desorption cycles).

**Figure 2:**
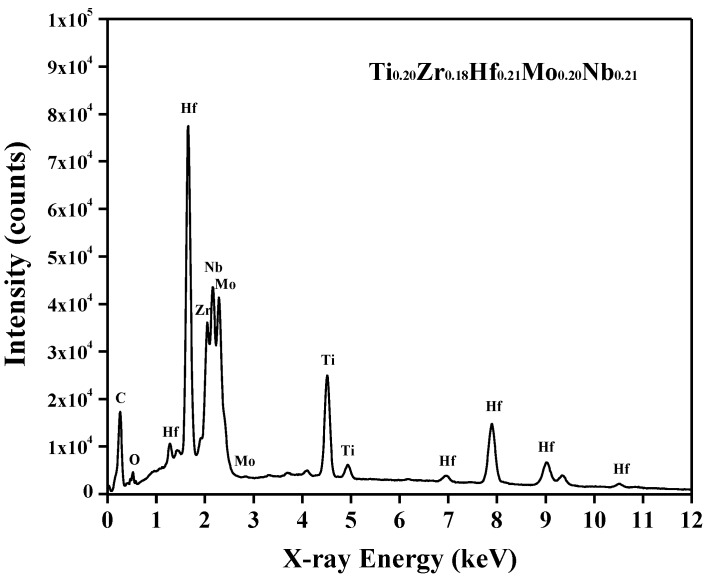
EDS spectrum of TiZrHfMoNb HEA alloy before hydrogenation.

Figure 1a,b show the XRD pattern, BF image and SAED pattern of the as-obtained TiZrHfMoNb alloy. As shown in Figure 1a, the XRD pattern contains four sharp diffraction peaks, which can be indexed as a BCC crystal structure with the lattice constant *a* = 0.3370(2) nm. No other diffraction peaks from intermetallic compounds or precipitates were found in the XRD patterns. Reconstruction of a series of SAED patterns taken from a grain further confirmed the BCC crystal structure of the TiZrHfMoNb alloy. One of the representative SAED patterns that was indexed as the <111> zone axis is shown in Figure 1b. As shown in Figure 2, the energy dispersive X-ray spectroscopy (EDS) confirmed that the composition of the alloy was Ti_0.20_Zr_0.18_Hf_0.21_Mo_0.20_Nb_0.21_, which was consistent with the designed equiatomic TiZrHfMoNb alloy. Only a tiny oxygen peak was found at an energy of around 0.52 keV, which indicated that this alloy was free of severe oxidation. The further EDS mapping results suggested that all Ti, Zr, Hf, Mo and Nb elements were distributed uniformly and no visible precipitate was found in this alloy.

The formation of a single-phase TiZrHfMoNb HEA was due to two major reasons. Each Ti, Zr and Hf pure metal has two phases, the *α* phase (HCP structure) and the *β* phase (BCC structure), which exist at RT and high temperature (above 800 °C), respectively. The addition of Mo and Nb alloying elements into the mixture of Ti, Zr and Hf metals is beneficial for the stabilization of the *β* phase of Ti, Zr and Hf at RT [12]. On the other hand, the atomic size difference (*δ*), the enthalpy of mixing (*△H*_mix_) and the entropy of mixing (*△S*_mix_) for TiZrHfMoNb alloy are 6.67%, −1.6 KJ/mol and 13.4 J/(K·mol), respectively, which conform to the design criterion of HEA summarized by Zhang et al. [16].

The TiZrHfMoNb powder was hydrogenated in a Sievert’s type apparatus with a base vacuum pressure that was greater than 1 × 10^−4^ Pa. Before hydrogenation, the TiZrHfMoNb powder was first thermally activated at 500 °C for 10 h in a 40 bar H_2_ environment, which was beneficial for promoting the hydrogen absorption of the alloy. The hydrogen was discharged by heating the TiZrHfMoNb hydride to 600 °C at a heating rate of 10 °C/min after activation. The hydrogenation experiment was conducted at 100 °C with a 5 bar H_2_ pressure for 1 h, which was followed by the cooling to RT to acquire the fully hydrogenated TiZrHfMoNb powder. Figure 1c shows the XRD pattern of TiZrHfMoNb hydride powder after five hydrogen absorption–desorption cycles. All five diffraction peaks can be indexed based on an FCC crystal structure with a lattice constant of *a* = 0.4590(4) nm. The crystal structure of the TiZrHfMoNb hydride was also confirmed by the reconstruction of SAED patterns taken from a same hydride particle. The representative SAED pattern indexed as <111> zone axis is shown in Figure 1d.

The above results on the structure of TiZrHfMoNb hydride were compared to the formation of a body-centered tetrahedral (BCT) structure of TiVZrNbHf hydride [22,23]. It is clear that the structure of TiZrHfMoNb HEA undergoes a phase transformation from a single BCC structure to a single FCC structure during the process of hydrogenation. The substitution of Mo for V in TiVZrNbHf HEA not only changes the crystal structure of its hydride from BCT to FCC, but also avoids the formation of two different dehydrogenation products with a BCC structure and slightly different lattice constants. Furthermore, this improves the phase transformation reversibility during the hydrogen absorption–desorption cycles [22]. It is interesting to note that no intermetallic or others hydride precipitate during the hydrogenation and dehydrogenation processes, which indicates that the disproportionation phenomenon of ZrCo-H system [10,11] did not occur in the TiZrHfMoNb-H system despite the mixing of more alloying elements in this HEA alloy. The formation of a single phase TiZrHfMoNb hydride is beneficial for improving the stabilization of hydrogenation performance, which results in the thorough release of H_2_ during desorption. Moreover, in contrast to the HCP structure of Ti, Zr and Hf pure metals, the cell volume of the BCC structure is larger than that of the HCP structure. Therefore, the formation of the BCC structure of the TiZrHfMoNb HEA is good for reducing its lattice expansion during hydrogenation and promoting the resistance to pulverization [12,13].

**Figure 3:**
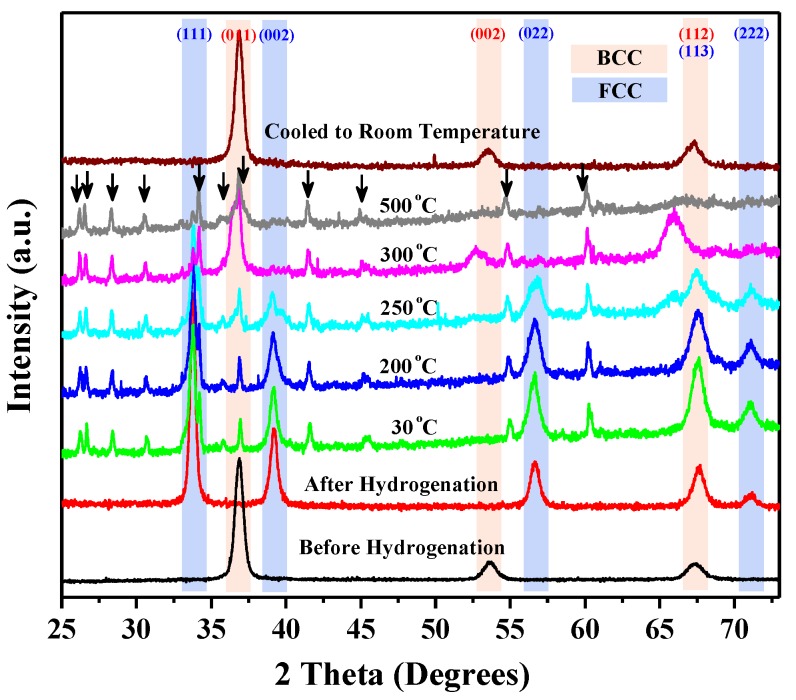
(Color online) The in situ heating XRD patterns of the TiZrHfMoNb hydride powder collected at temperatures from RT to 500 °C and the XRD pattern acquired after the sample was cooled down to RT.

The most notable hydrogenation performance of TiZrHfMoNb alloy was related to its reversible single-phase transformation property during a hydrogen absorption–desorption cycle. The in situ XRD test was explored to verify the reversibility of phase transformation of the TiZrHfMoNb hydride during hydrogen desorption. Figure 3 shows the in situ heating XRD patterns of TiZrHfMoNb hydride powder collected at RT, 30 °C, 200 °C, 250 °C, 300 °C and 500 °C. For comparison, the XRD pattern of the original TiZrHfMoNb powder is also presented in Figure 3. The diffraction peaks labeled by arrows were due to the chamber window made of the X-ray transparent material Kapton. The in situ heating XRD results demonstrated that the TiZrHfMoNb hydride powder still had a FCC structure at temperatures below 250 °C. A subtle lattice expansion from 0.4590(4) nm to 0.4616(3) nm was observed in the in situ heating XRD patterns when the sample was heated from RT to 250 °C. Once the temperature reaches 300 °C, the TiZrHfMoNb hydride started to desorb and transform back to the BCC structure of the original TiZrHfMoNb alloy. After heating to 500 °C, the sample was cooled to RT. The XRD pattern collected at RT confirmed that the FCC TiZrHfMoNb hydride was transformed back to the BCC TiZrHfMoNb alloy.

**Figure 4:**
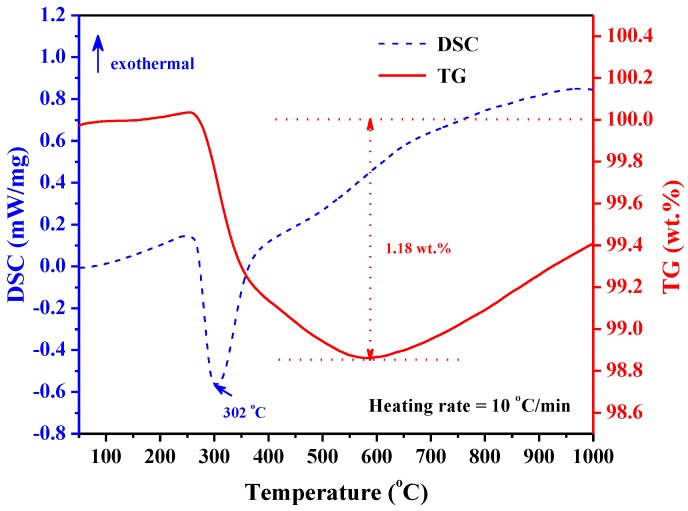
(Color online) The TDS spectra of the TiZrHfMoNb hydride powder.

The thermal stabilization of the TiZrHfMoNb hydride was further measured by the TDS method. Figure 4 shows the heat flow and thermal gravimetric (TG) curves during the TDS test. Only one peak was observed at 302 °C in the whole differential scanning calorimetry (DSC) curve, which indicated that the desorption of TiZrHfMoNb hydride is an endothermic process and would be completely finished in one step. In the TG curve, the weight of the TiZrHfMoNb hydride drops rapidly at around 300 °C and starts to increase from 590 °C, which might be due to the oxidation of hydride powders. During the hydrogen desorption, the maximum weight loss is 1.18 wt.%. It is concluded that the TiZrHfMoNb hydride desorbs hydrogen very fast at 302 °C, which is consistent with the in situ heating XRD results. The hydrogen storage capacity of TiZrHfMoNb HEA is 1.18 wt.%, which could be expressed as the following chemical formula TiZrHfMoNbH_6.05_. It should be noted that the reported hydrogen storage capacity of 1.18 wt.% for TiZrHfMoNb alloy was obtained and was found to be stable at RT and in the atmospheric environment, which was contrary to that of 2.7 wt.% for TiZrHfVNb alloy obtained in the high pressure hydrogen environment (up to 53 bar) [22]. The above-mentioned results demonstrated that the TiZrHfMoNb HEA has both advantages of good thermal stability and high storage capacity of hydrogen in a solid phase.

**Figure 5:**
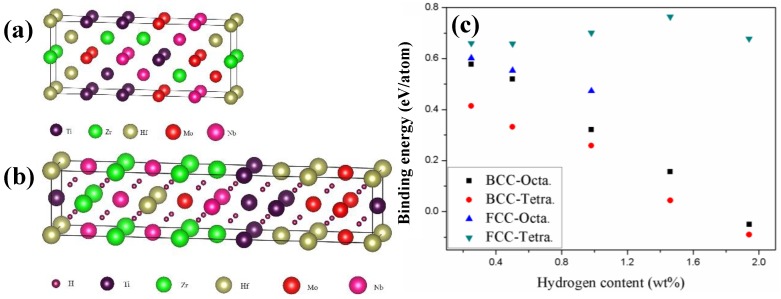
(Color online) The BCC atomic model before hydrogenation (**a**) and the hydrogenated FCC atomic model (**b**). (**c**) Variation of the binding energy for the BCC and FCC hydrogenated TiZrHfMoNb with the H content. (Octa.: octahedral occupation for H; Tetra.: Tetrahedral occupation for H.)

To explore the origin of the BCC-to-FCC reversible phase transformation during hydrogenation and dehydrogenation, the phase stability of BCC and FCC TiZrHfMoNb HEAs before and after hydrogenation was investigated by DFT calculations. For the BCC TiZrHfMoNb HEA, the lattice constant was calculated to be 0.336 nm, which is consistent with the experimental value of 0.337 nm. Figure 5a,b show the BCC and FCC atomic models plotted using the VESTA program [30] for the alloy before and after hydrogenation, respectively. The BCC models of TiZrHfMoNb alloy were randomly generated by a Python program and the model with the lowest energy was selected as the computational model. The FCC models with H atoms occupying the tetrahedral or octahedral sites were also constructed by the Python program and the selected computational model had the lowest energy. The lattice constants for the computation models were the consistent with the XRD results. For the pure TiZrHfMoNb HEA, it was shown that the BCC phase is energetically more favorable than the FCC phase, since the total energy of the BCC phase is 0.25 eV/atom lower than that of the FCC phase. As for the hydrogenated TiZrHfMoNb, both octahedral and tetrahedral occupations for H with different contents were considered. The hydrogen binding energy (*E*_B_) was calculated to compare the phase stability of hydrides and the equation was defined as follows [31]:

$$E_{B\;}=-\frac{1}{x}\left[E_{tot}\left(MH_{x}\right)-E_{tot}\left(M\right)-\frac{x}{2}E\left(H_{2}\right)\right]$$

where *E_tot_* (*MH_x_*) is the total energy of the TiZrHfMoNb alloy with *x* hydrogen concentration, *E_tot_* (*M*) is the total energy of the pure alloy without hydrogen and *E*(*H*_2_) is the total energy of the hydrogen molecule. The binding energy indicates the interaction between the hydrogen and the TiZrHfMoNb alloy, which is generally positive. A larger value indicates a stronger interaction. The binding energies for the hydrogenated TiZrHfMoNb with both BCC and FCC phases were calculated and are shown in Figure 5c. One interesting finding is that the hydrogenated FCC TiZrHfMoNb is always more preferable than the BCC phase in terms of energy as indicated by the larger binding energy, which is consistent with our experimental results. 

The above-mentioned theoretical results indicate that the hydrogenation induced a BCC-to-FCC phase transformation, which is an exothermic process with positive binding energy. It is also noted that in the BCC phase, the hydrogen prefers to occupy the octahedral interstitial sites, whereas the tetrahedral interstitial sites are preferable in the FCC phase. Miraglia et al. [32] investigated the phase transformation in a Ti-V-Cr alloy and they also found that after hydrogenation, the FCC phase is more stable than the BCC phase and the hydrogen occupies the octahedral and tetrahedral sites in the BCC and FCC phases, respectively. Based on the optimized structures for hydrogenated FCC TiZrHfMoNb, a detailed structural analysis was performed. It turns out that during hydrogenation and dehydrogenation, no other metal hydride and intermetallic is formed, which is consistent with experimental observations.

## 4. Conclusions

In conclusion, a novel TiZrHfMoNb high-entropy alloy was designed and synthesized by the arc-melting method. The TiZrHfMoNb alloy has a single-phase BCC crystal structure and undergoes a phase transformation to a FCC crystal structure after hydrogenation. The TiZrHfMoNb hydride starts to desorb hydrogen at around 302 °C. The TiZrHfMoNb HEA alloy could be used for hydrogen storage in a solid phase and thus, can be a candidate material for solar thermal energy storage due to its fantastic properties of good thermal stability and reversible single-phase transformation during the hydrogen absorption–desorption cycles.
